# The Comparison of Dexmedetomidine to Dexamethasone as Adjuvants to Bupivacaine in Ultrasound-Guided Infraclavicular Brachial Plexus Block in Upper Limb Surgeries

**DOI:** 10.7759/cureus.41668

**Published:** 2023-07-10

**Authors:** Swathy S Iyengar, Anshu Pangotra, Kumar Abhishek, Nitesh Sinha, Natesh S Rao, Vinod K Singh, Jay Prakash

**Affiliations:** 1 Department of Neuroanesthesia, People Tree Institute of Neurosciences, Bengaluru, IND; 2 Department of Anesthesiology, Acharya Shri Chander College of Medical Sciences and Hospital, Jammu, IND; 3 Department of Trauma Critical Care, Rajendra Institute of Medical Sciences, Ranchi, IND; 4 Department of Anesthesiology, Rajendra Institute of Medical Sciences, Ranchi, IND; 5 Department of Anesthesia, Vydehi Institute of Medical Sciences and Research Centre, Bengaluru, IND; 6 Department of Critical Care Medicine, Sir Ganga Ram Hospital, New Delhi, IND; 7 Department of Critical Care Medicine, Rajendra Institute of Medical Sciences, Ranchi, IND

**Keywords:** adjuvants, dexmedetomidine, dexamethasone, bupivacaine, infraclavicular brachial plexus

## Abstract

Background

The clinical utility of adjuvants with local anesthesia produces an excellent nerve block with prolonged duration and faster onset. Brachial plexus block is widely used nowadays in patients undergoing upper limb surgery There are several approaches to achieve brachial plexus block such as interscalene, supraclavicular, infraclavicular, and axillary. The objective of this study is to compare the effectiveness of dexamethasone to dexmedetomidine as adjuvants to bupivacaine in patients undergoing ultrasound-guided infraclavicular brachial plexus (USG-ICBP) block.

Methods

A randomized, prospective, double-blind study was undertaken on the patients posted for upper limb surgeries under ultrasound-guided infraclavicular brachial plexus block. Sixty patients with the American Society of Anesthesiologists (ASA) classes I and II were randomly allocated into two groups. Group A received 25 mL of 0.5% bupivacaine and 1.5 mL (6 mg) of dexamethasone, and group B received 25 mL of 0.5% bupivacaine and 0.75 mL (75 mcg) of dexmedetomidine along with 0.75 mL of 0.9% normal saline (NS). Student’s t test or Mann-Whitney test and chi-square test were used for statistical analysis.

Results

The onset of sensory block was significantly faster in the patients in group B as compared to the patients in group A. In terms of the duration of the block, sensory and motor blocks were maintained for a significantly longer duration in the group A patients as compared to those in group B. Moreover, the duration of postoperative analgesia was significantly longer-lasting in the group A patients. In terms of adverse effects, procedure-related complications such as the failure of the block and inadequate block were comparable across the groups. However, drug-related adverse effects were significantly more common in group B.

Conclusion

As compared to 75 mcg of dexmedetomidine, the addition of 6 mg of dexamethasone as adjuvant to 25 mL of 0.5% bupivacaine resulted in significantly longer-lasting sensory and motor blocks, postoperative analgesia, and a delayed time for first rescue analgesia without increasing undue adverse effects. Dexmedetomidine use is associated with more sedation as compared to dexamethasone.

## Introduction

Brachial plexus block is a frequent technique used nowadays for procedures on the upper extremities [1]. An experienced anesthetist can safely perform the procedure with low rates of complications while simultaneously avoiding the systemic complications associated with general anesthesia. The supraclavicular approach for brachial plexus block is the most popular one, although there are different approaches such as interscalene, infraclavicular, and axillary to perform successful brachial plexus block [2].

Other adjuvants are routinely added to the local anesthetic (LA) to increase the effectiveness of the block. The most often used adjuvants are dexamethasone (long-acting glucocorticoid) and dexmedetomidine (centrally acting alpha-adrenergic agonist). Many previous studies have examined the efficacy of these adjuvants in subjects undergoing supraclavicular brachial plexus block [2,3].

For regional anesthesia to the upper limb, both the supraclavicular and infraclavicular blocks are consistently and reliably employed. When compared to other brachial plexus blocks, they have been demonstrated to be more effective in a number of respects such as quicker procedure time, less supplemental anesthesia, faster onset, and fewer complications [4]. However, studies comparing their efficacy in patients undergoing infraclavicular block are rare.

The current study aims to reduce the gap by directly comparing the efficacy of dexamethasone to dexmedetomidine as adjuvant to bupivacaine in patients having ultrasound-guided infraclavicular brachial plexus (USG-ICBP) block.

The primary objective of our study was to determine when motor and sensory blocks would start to occur and how long they would last. Secondary objectives included analgesia duration, hemodynamic changes, post-procedure sedation, and any other complications related to the procedure or drug side effects.

## Materials and methods

This is a prospective, randomized, double-blind study. This study was carried out over the course of a year, from October 2015 to October 2016, and approval was obtained from the Vydehi Institutional Ethics Committee via letter number VIEC/2015/APP/096 with ethical committee (EC) registration number ECR/747/Inst/KA/2015 dated October 30, 2015. A total of 60 American Society of Anesthesiologists (ASA) grade I and II patients between the ages of 18 and 60 who were undergoing elective upper limb surgery under USG-ICBP block participated in the study after providing written informed consent. According to the “Consolidated Standards of Reporting Trials (CONSORT) guidelines,” which improve the reporting of randomized clinical trials, every aspect of the study was assessed.

Participation in the study was not permitted for the patients who were ineligible for brachial plexus block due to conditions such as severe lung disease, preexisting neuropathic conditions, vascular insufficiency affecting the surgical limb, local site infections, coagulopathy, uncontrolled systemic disease, a complication related to blocks, or hypersensitivity to study drugs. The patients taking adrenergic antagonists and beta-blockers, two drugs that have an impact on the autonomic nervous system, were also excluded from the trial.

In order to ensure the concealment of the allocation sequence, 60 patients were randomly split into two groups (group A, n = 30; group B, n = 30) using computer-generated randomization numbers and the sealed envelope method. In the operating room, the anesthesiologist, who was not involved in the study, opened the package and prepared the medication. The block was carried out; the principal investigator monitored and recorded the parameters while being drug-blinded. As a result, the drug was blinded from the patients, as well as the attending anesthesiologist.

The patients were divided into two groups at random: group A received 25 mL of 0.5% bupivacaine and 1.5 mL (6 mg) of dexamethasone (total volume = 26.5 mL). Group B received 25 mL of 0.5% bupivacaine and 0.75 mL (75 mcg) of dexmedetomidine along with 0.75 mL of 0.9% normal saline (NS) (total volume = 26.5 mL).

Prior to surgery, each patient had a complete preanesthetic evaluation and sufficient optimization. The procedure was carried out in an operating room while being monitored according to conventional ASA protocols, which included using a pulse oximeter, a noninvasive blood pressure (NIBP) monitoring, and an electrocardiogram (ECG) in a non-procedural upper leg. A 20-gauge intravenous (IV) cannula was used to secure intravenous (IV) access in the opposite arm.

Midazolam (1 mg) and glycopyrrolate (0.2 mg) were administered IV to the patients as premedication just prior to the surgery. The supine position was employed for the patients. The arms were either by the side of the body or 90° abducted, and the head was tilted to the opposite side to achieve maximum exposure. An anesthetist who was not involved in the trial gave the block while upholding stringent asepsis. The brachial plexus was located using a linear transducer (8-13 MHz) probe and a portable ultrasound equipment, Siemens Acuson Freestyle™ (Siemens Healthcare, Camberley, United Kingdom). The brachial plexus was perceived as having a characteristic “cluster of grapes” look with the probe positioned beneath the clavicle and medial to the coracoid process (hypoechoic circular shadows with hyperechoic outer ring). To administer the local anesthetic combination, a 22-gauge beveled needle (Stimuplex Ultra, B. Braun Melsungen AG, Melsungen, Germany) was utilized. A negative aspiration technique was utilized to avoid accidentally injecting the local anesthetic into a blood artery.

At zero, five, 10, 15, 30, 45, 60, 90, and 120 minutes after the LA combination was effectively administered, sensory and motor blocks were assessed. After the block was finished, evaluations were conducted every six hours for the next 24 hours. The extent of sensory blockade in the radial, ulnar, median, and musculocutaneous nerve regions was assessed by pinprick testing: grade 0 (no sensory block {sharp pain felt}), grade 1 (analgesia {dull feeling felt}), and grade 2 (anesthesia {no sensation felt}) were the three potential results for the test [5]. The patient was deemed to have achieved a grade 2 along all the nerve areas on a scale of 3. The time between LA injection and the attainment of complete sensory block was used to define the time for sensory blockade to begin. If anesthesia was not present in two or more nerve distributions within 30 minutes of injection, the block was considered to have failed, and those subjects were removed from the analysis.

According to the modified Bromage scale [6], which is graded from 0 (normal motor function with full flexion and extension of the elbow, wrist, and fingers) to 2 (complete motor block), motor block was evaluated by testing for thumb abduction (radial nerve), thumb adduction (ulnar nerve), thumb opposition (median nerve), and elbow flexion (musculocutaneous nerve). When patients received grade 2 along each of the four nerve regions, they were deemed to have completely blocked their motor function. The interval between LA injection and the beginning of a complete motor block was used to establish the timing of motor block onset.

The block was regarded successful when at least two of the four nerve regions (ulnar, radial, median, and musculocutaneous) were successfully blocked for both sensory and motor components. If the block had been deemed insufficient for any reason after 30 minutes, general anesthesia was given to the patient, and they were then removed from the trial.

The average duration of surgery was approximately two hours and 30 minutes. Every 60 minutes until the pinprick response was felt in at least three major nerve areas, the sensory blocking was evaluated, and the period from assessment to pinprick feeling was recorded as the duration of analgesia. Until total muscle power was recovered in at least two major nerve distributions, the motor blockade was evaluated every 60 minutes, and the length of time between assessments was recorded as the duration of the motor blockade.

The verbal response score (VRS) was used to determine the intensity of postoperative pain [7]. When the patients reported a VRS of 3 or higher, rescue analgesia (diclofenac 1-1.5 mg/kg IV in 100 mL NS over 15 minutes followed by tramadol 1 mg/kg IV, if needed) was given. The time to first rescue analgesia was calculated as the time from the start of total sensory block to the first analgesic request.

The modified Ramsay Sedation Score was used to determine the patient’s sedation score [8]. Additionally, blood pressure, pulse, peripheral oxygen saturation (SpO_2_), and postoperative nausea and vomiting were also noted.

Statistical analysis

In order to do the statistical analysis, Stata 11 (StataCorp LLC, College Station, TX) was used. The Shapiro-Wilk test was used to gauge normality. The Mann-Whitney test or Student’s t test was used, as applicable, to examine whether there was a difference between the means of the two groups for continuous variables. Chi-square test was used to examine the categorical variables, which have been reported as frequency and percentage. A P-value of <0.05 was considered statistically significant.

## Results

**Figure 1:**
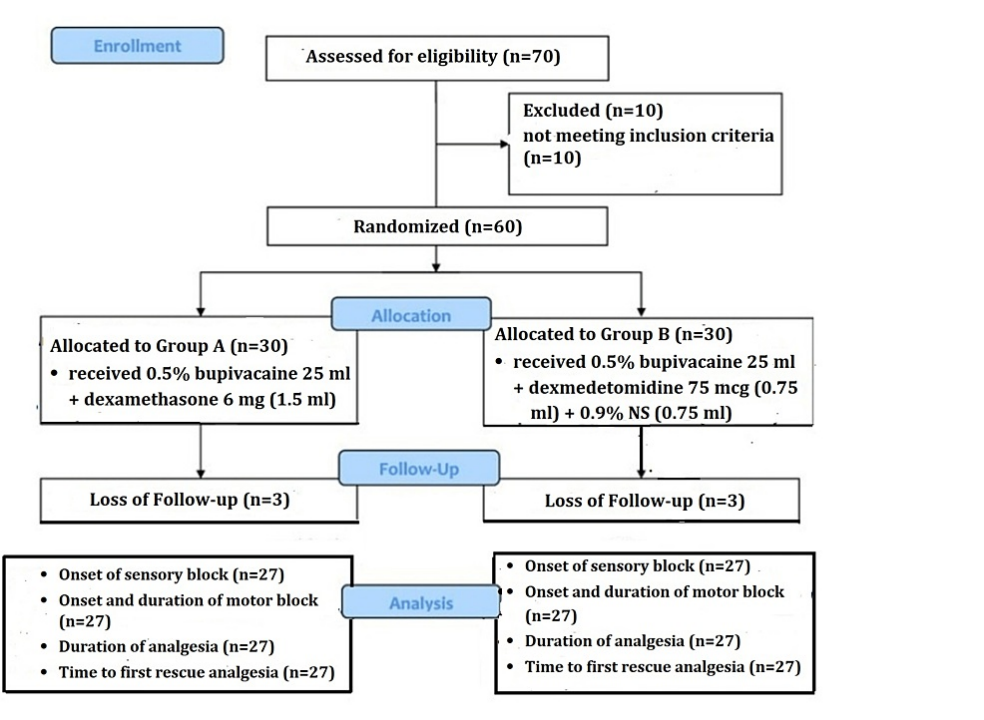
CONSORT flow diagram CONSORT, Consolidated Standards of Reporting Trials; NS, normal saline

The participating patients with successful ICBP blocks completed the study (Figure 1). Regarding baseline parameters such as patient demographics and ASA grading, the patients in the two groups were compared (Table 1).

**Table 1:** Demographic characteristics of the patients Group A: bupivacaine + dexamethasone; group B: bupivacaine + dexmedetomidine. All values were expressed as mean ± SD or number of subjects (%) ASA: American Society of Anesthesiologists

	Group A	Group B	P-value
Age (years)	37.17 ± 13.32	33.77 ± 12.13	0.33
Gender			
Male	23 (85.2%)	19 (70.4%)	0.19
Female	4 (14.8%)	8 (29.6%)	
Weight (kg)	60.57 ± 9.59	59.30 ± 6.96	0.58
Height (cm)	157.8 ± 8.07	155.63 ± 5.76	0.26
ASA grade			
Grade I	22 (81.5%)	17 (63%)	0.12
Grade II	5 (18.5%)	10 (37%)	
Duration of surgery (minutes)	147.30 ± 55.19	142.90 ± 58.81	0.78

The patients in group B experienced sensory block much earlier than the patients in group A. In group B, motor block developed more quickly, and the patients in group A were comparable to those in group B. The patients in group A sustained both sensory and motor blocks for noticeably longer periods of time than the patients in group B, according to the duration of the block. Moreover, the duration of postoperative analgesia was significantly longer-lasting in the group A patients. The time to first rescue drug was significantly shorter in the group B patients (Table 2).

**Table 2:** Block characteristics Group A: bupivacaine + dexamethasone; group B: bupivacaine + dexmedetomidine. All values were expressed as mean ± SD or number of subjects (%)

	Group A	Group B	P-value
Time taken to perform the block (minutes)	10.24 ± 2.46	11.24 ± 2.86	0.174
Onset of complete sensory block (minutes)	13.23 ± 3.46	10.87 ± 2.22	0.004
Onset of complete motor block (minutes)	18.53 ± 5.59	16.30 ± 3.62	0.087
Duration of sensory block (minutes)	1091.79 ± 151.29	734 ± 140.87	<0.0001
Duration of motor block (minutes)	979.29 ± 141.47	636 ± 148.94	<0.0001
Time to first rescue analgesia (minutes)	990 ± 170.23	518 ± 150.12	<0.0001
Duration of analgesia (minutes)	942.21 ± 168.41	591.10 ± 150.29	<0.0001

Concerning negative impacts, procedure-related issues such as block failure and insufficient block were equivalent across the groups. Complications related to the block (such as failed block, inadequate or patchy block, neuropathy, vascular puncture, and pain during or after the block in the localized area) were excluded from the study in accordance with our protocol. Hence, we analyzed the effect of both adjuvants in 27 patients in both groups (Table 3).

**Table 3:** Ramsay Sedation Score, adverse effects, and complications Group A: bupivacaine + dexamethasone; group B: bupivacaine + dexmedetomidine. All values are expressed as mean ± SD or number of subjects (%) ^£,¥,β,€^Complications of the block were excluded from the study

	Group A	Group B	P-value
Ramsay Sedation Score			
2	27 (100%)	9 (33%)	<0.001
3	0 (0%)	9 (33%)	
4	0 (0%)	8 (30%)	
5	0 (0%)	1 (3%)	
Adverse effects of the drugs			
None	25 (93%)	15 (60%)	<0.001
Bradycardia	0 (0%)	1 (3%)	
Headache	2 (7%)	0 (0%)	
Nausea and vomiting	0 (0%)	1 (3%)	
Sedation	0 (0%)	8 (27%)	
Sedation and bradycardia	0 (0%)	1 (3%)	
Sedation and hypotension	0 (0%)	1 (3%)	
Complications of the block			
Nil	27 (90%)	27 (90%)	1.00
^£^Failed block	1 (3%)	0 (0%)	
Inadequate block	0 (0%)	1 (3%)	
^¥^Neuropathy	1 (3%)	0 (0%)	
^β^Pain during block	1 (3%)	0 (0%)	
^€^Vascular puncture	0 (0%)	2 (6%)	

We observed that group B had more adverse effects. The group B patients experienced drowsiness not only at higher rates but also at deeper depths, with one patient receiving a Ramsay Drowsiness Score of 5. The patients in group B experienced significant bradycardia that lasted for up to two hours; however, the group A patients did not experience a similar reduction in heart rate. Systolic and diastolic blood pressures in both groups were similar, and only one member of group B experienced clinically significant hypotension. In neither of the groups, there was a clinically significant decrease in oxygen saturation.

In our study, there were no cases of pneumothorax, Horner’s syndrome, phrenic nerve palsy, vascular problems, or postoperative complications such as hematoma. However, two patients in the dexmedetomidine group experienced neurological side effects because a vascular puncture was unintentionally made when placing the block. Additionally, one patient receiving dexamethasone experienced a failed block that required conversion to general anesthesia. A single patient who underwent surgery without incident experienced prolonged sensory and motor impairments with tingling and paresthesia over the distal arm, forearm, and hand in the early postoperative period. These symptoms persisted for more than 48 hours. Brachial plexus injury was excluded since only specific nerves affecting all the cords were injured, not a single cord as a whole extended tourniquet usage throughout the perioperative phase was identified as the culprit, and further nerve conduction investigations supported this theory. Further nerve conduction measurements later indicated that the cause was prolonged tourniquet time during the intraoperative phase.

## Discussion

The patients having procedures on their upper limbs are increasingly using brachial plexus blocks. Portable ultrasonography is now commonly used in routine anesthetic procedures [9]. Further reduction in the amount of LA to be injected is made possible by the capacity to accurately implant the LA in the perineural area. A previous study has proven that using ultrasound to deliver an infraclavicular block results in better outcomes and fewer problems [10]. As a result, it improves not only the efficacy and results of the surgery but also its safety.

There has been a lot of attention recently on the infraclavicular approach [11]. In contrast to other techniques, it does not only prevent consequences including pneumothorax, phrenic nerve damage, and unintentional stellate ganglion block [12]. Additionally, it can effectively anesthetize the axillary, musculocutaneous, radial, ulnar, and median nerves in the upper limb [13]. The search for an adjuvant that can assist in extending the LA’s activity without also adding to the difficulties has been ongoing [14]. Numerous drugs, including neostigmine, tramadol, dexamethasone, clonidine, and dexmedetomidine, have been explored [15].

In this study, dexamethasone and dexmedetomidine, two adjuvants, were assessed for their efficacy and safety. Both adjuvants have previously undergone extensive research [15-17]. However, the supraclavicular technique has been used in the majority of these trials to block the brachial plexus [18]. We found that adding 6 mg of dexamethasone as an adjuvant to 25 mL of 0.5% bupivacaine produced sensory and motor blocks that lasted noticeably longer than 75 mcg of dexmedetomidine, postoperative analgesia, and delayed time for the first rescue analgesic without causing unneeded adverse effects.

In our study, the mean time of onset of sensory block was faster in the dexmedetomidine group (13.23 ± 3.46 minutes in the dexamethasone group versus 10.87 ± 2.22 minutes in the dexmedetomidine group). Similar findings were reported by Aliste et al. (21.7 ± 5.6 minutes and 19.5 ± 5.5 minutes, respectively; p = 0.040) [19]. Yaghoobi et al. reported a higher mean onset of sensory block in the dexmedetomidine group, though the difference was not significant in their study (13.6 ± 6.0 and 15.6 ± 6.7 minutes, respectively; p = 0.75) [20].

There was no difference in terms of the onset of motor block in our study (18.53 ± 5.59 minutes and 16.30 ± 3.62 minutes, respectively; p = 0.087), which is consistent with 13.0 ± 5.4 minutes and 13.8 ± 7.2 minutes, respectively (p = 0.924) [20]. Ghazaly et al. [21] reported a mean duration of sensory of 14.55 hours and motor block of 15.6 hours in the patients undergoing ICBP with dexmedetomidine (100 mcg) plus levobupivacaine, which was similar to our findings.

We found that the sensory block persisted for a longer duration in the dexamethasone group (18.17 ± 2.51 hours in the dexamethasone group versus 12.23 ± 2.33 hours in the dexmedetomidine group). However, the findings of Yaghoobi et al. differed significantly; not only did they report a much shorter duration of sensory block of ~3-4 hours, but also the block persisted longer in the dexmedetomidine group (217.1 ± 98.9 minutes versus 278.81 ± 81.0 minutes; p = 0.046) [20]. Dexamethasone was also found to provide a motor block for significantly longer duration (16.31 ± 2.35 versus 10.6 ± 2.48; p < 0.0001) in our study, which is corroborated by 17.4 ± 4.0 hours versus 14.3 ± 3.0 hours (p < 0.001) [19]. These findings were again at odds with those of much shorter duration of action (249.1 ± 106.6 versus 284.4 ± 77.9; p = 0.212) [20].

Another study conducted by Esmaoglu et al. [22] in the patients undergoing infraclavicular brachial plexus block with dexmedetomidine plus levobupivacaine reported a median duration of sensory and motor blocks of 14.78 hours and 12.88 hours, respectively. Some of the reasons for such discordant results in the onset and duration of the block could be due to differences in the drugs used, the doses of the drugs, interpatient variations in the study population, variations in the anatomy of individual patients, and differences in the techniques employed by different anesthesiologists.

The time to first rescue analgesia was longer for the dexamethasone group in our study (990 ± 170.23 minutes versus 518 ± 150.12 minutes; p ≤ 0.0001). However, there was no difference in the time to first rescue analgesia (323.0 ± 70.6 minutes versus 324.8 ± 57.2 minutes, respectively; p = 0.86) [20]. The duration of analgesia was higher for the dexamethasone group in both our study (15.7 ± 2.8 hours and 9.85 ± 2.5 hours, respectively; p < 0.0001) and another study (22.2 ± 3.6 hours and 16.9 ± 3.9 hours, respectively; p < 0.001) [19]. Although most of the results of our study are consistent with previous studies, the differences might be explained by the differences in the concentrations of the study drugs.

In terms of the adverse effects, a fall in heart rate and mean arterial pressure was noticed more commonly in the dexmedetomidine group in our study, as well as those of other studies (p = 0.006 [20] and p = 0.001 [19]). Dexmedetomidine also resulted in an increased level of sedation postoperatively, as was also found by other studies [18,19].

We are aware of our study’s limitations. Firstly, we used fixed doses of drugs instead of using doses adjusted to the body weight. Furthermore, since the equipotent doses of the drugs are not well established, the doses mentioned in the previous studies were used. For instance, in the previous studies, the dose of dexamethasone for achieving peripheral nerve blocks varies between 2 and 10 mg [23]. Similarly, most studies have used doses of dexmedetomidine varying between 50 mcg and 150 mcg [24]. We sought to take the middle ground and use doses that were somewhere around the middle. Secondly, the sample size was relatively small. Thirdly, our observation period was limited to 24 hours after the surgery. Therefore, any adverse effects that might have occurred after that period could not be studied. Finally, our study included a wide variety of procedures being performed by various surgeons. Differences between the groups could arise due to differences in the type of procedures, as well as differences in the operating surgeon’s skills.

## Conclusions

When compared to adding dexmedetomidine as an adjuvant, adding 6 mg of dexamethasone as an adjuvant to 25 mL of 0.5% bupivacaine resulted in a significantly longer-lasting sensory and motor blocks, postoperative analgesia, and delayed time for the first rescue analgesia without increasing unwarranted adverse effects. Dexamethasone is a superior adjuvant to local anesthetic for infraclavicular brachial plexus block because dexmedetomidine administration is associated with higher intra- and post-procedure drowsiness than dexamethasone.
